# 非小细胞肺癌的*BRAF*基因突变及其临床意义

**DOI:** 10.3779/j.issn.1009-3419.2012.03.09

**Published:** 2012-03-20

**Authors:** 志敏 黄, 一龙 吴

**Affiliations:** 510080 广州，广东省肺癌研究所，广东省人民医院，广东省医学科学院 Division of Pulmonary Oncology, Guangdong Lung Cancer Institute, Guangdong General Hospital and Guangdong Academy of Medical Sciences, Guangzhou 510080, China

**Keywords:** *BRAF*突变, 肺肿瘤, 靶向治疗, *BRAF* mutation, Lung neoplasms, Targeted therapy

## Abstract

*BRAF*基因是一个驱动基因，可能是靶向治疗非小细胞肺癌的一个靶点。本文就*BRAF*基因的结构、表达、信号通路调节及研究热点、与肿瘤发生的关系尤其是与非小细胞肺癌的靶向治疗关系加以阐述。

## Abstract (en)

*BRAF* mutations have been found to be a driver mutation and maybe a therapy target in patients with non-small cell lung cancer. This article reviews the current understanding of *BRAF* gene, its structure, expression, the signal pathway, as well as its relationship with cancer especially the targeted therapies for non-small cell lung cancer.

*BRAF*基因激活突变可发生于多种肿瘤及癌细胞系中，尤其常见于恶性黑色素瘤。近年来研究提示*BRAF*基因激活突变可能与肺癌的发生发展及治疗预后有密切的关系。本文就*BRAF*基因的结构、表达、信号通路调节及研究热点、与肿瘤发生的关系尤其是与非小细胞肺癌（non-small cell lung cancer, NSCLC）的靶向治疗关系加以阐述。

## 1 *BRAF*基因及家族

*BRAF*基因，1988年由Ikawa等^[1]^首先在人类尤文氏肉瘤中发现并克隆确认，是一个能转染NIH3T3细胞且有活性的DNA序列，因其与CRAF和ARAF具有相当高的同源性，故称其为BRAF。位于染色体7q34的*BRAF*基因编码丝氨酸/苏氨酸蛋白激酶，是RAF家族成员之一，该家族还包括ARAF、CRAF，三者的编码产物均存在于细胞间质中，其中ARAF蛋白主要位于泌尿生殖器官，BRAF主要位于神经及睾丸组织，CRAF在人体内广泛分布。BRAF蛋白由783个氨基酸组成，从N端到C端依次为CR1、CR2和CR3三个保守区。其中CR1区由RAS蛋白结合区和富含半胱氨酸区组成，这两个区域均可与RAS结合；CR2富含丝氨酸/苏氨酸，为调节磷酸化RAF激酶活性；CR3区为ATP结合位点和激活区，含有酪氨酸和丝氨酸残基及有多个磷酸化位点，其中T598和S601最为重要，这两个位点同时磷酸化后可激活BRAF蛋白和诱导性激活ERK^[2]^，而这两个位点氨基酸的置换将导致BRAF蛋白持续性活化。

## 2 BRAF与RAS-RAF-MEK-ERK信号通路

BRAF蛋白与KRAS蛋白同为RAS-RAF-MEK-ERK信号通路中上游调节因子^[3]^，在MAPK/ERK信号通路中起着举足轻重的作用。RAS蛋白属于小GTP结合蛋白，与三磷酸鸟苷GTP及二磷酸鸟苷GDP有很强的亲和性，RAS-GTP为RAS的活化形式，RAS-GDP为非活化形式，二者能够相互转化，对细胞分化、增殖起调节作用^[4]^。酪氨酸激酶受体与生长因子结合，能使酪氨酸蛋白激酶受体二聚化及残基磷酸化，促使衔接分子Grb2与SOS结合，细胞外信息经此途径被转导至细胞内。SOS通过使RAS-GDP转化RAS-GTP从而活化RAS激酶活性。RAS-GTP复合物可调节一系列细胞进程，如增殖、生长和分化^[5]^。RAS可以通过激活至少10条下游反应通路途径而发挥作用。其中研究比较透彻的是RAF-MEK-ERK通路（经典MAPK通路）。RAS-GTP与其下游效应分子RAF结合，使RAF磷酸化并激活，从而激活了RAF下游信号转导MAPK级联途径。RAF蛋白磷酸化并激活下游底物MEK，而MEK磷酸化并激活下游底物ERK，进而调节细胞内的生物学过程^[6]^。其中MEK具有磷酸化苏氨酸/酪氨酸残基的双特异功能，ERK为富含脯氨酸的丝氨酸/苏氨酸蛋白激酶。

## 3 BRAF的分子变异

目前有超过40种*BRAF*突变被识别^[7]^，其中最主要有两种类型，80%突变发生于激酶功能域的V600E（即外显子15），20%突变位于外显子11的甘氨酸环。以上两种突变均可使BRAF激酶活性提高。研究证实V600E突变后能够产生类似于T598和S601两个位点磷酸化的作用，使BRAF蛋白持续激活，激活后的BRAF通过下游MEK/ERK传导通路刺激细胞的增殖和分化。其它一些发生率较低的BRAF基因突变多集中于外显子11甘氨酸环的突变如R461I、I462S、G463E、G463V、G465A、G465E、G465V、G468A、G468E、N580S、E585K、D593V、F594L、G595R、L596V、T598I、V600D、V600E、V600K、V600R、K601E、A727V等，也可通过直接激活BRAF而激活MEK/ERK传导通路^[2]^。

## 4 *BRAF*突变与肿瘤

肿瘤的发生与介导细胞增殖、分化及凋亡的一些关键基因的突变有密切关系，RAS信号转导通路的激活与许多肿瘤的发生有关。*BRAF*基因突变可通过两种途径致病，一是因遗传而导致先天性缺陷的途径，二是后天获得致癌基因而导致肿瘤的途径。BRAF蛋白与KRAS蛋白同为RAS-RAF-MEK-ERK信号通路中上游调节因子^[3]^。BRAF在MAP/ERKs信号通路中起着举足轻重的作用，BRAF蛋白位于MEK/ERK途径的入口处，它将细胞表面的受体和RAS蛋白通过MEK和ERK与核内的转录因子相连接，影响细胞分化、分裂、增殖、生长^[3]^。BRAF的持续激活可引起MEK/ERK信号转导紊乱，导致细胞过度增殖、恶变。*BRAF*基因突变最先在黑色素瘤患者中检测到，在黑色素瘤、甲状腺瘤中突变率较多（约80%），而在淋巴瘤、直肠癌、肝癌、乳腺癌、卵巢癌及肺癌中突变率较少^[7]^。*BRAF*突变以T1799A点突变即V600E最为常见，T1799A点突变是外显子15上第1, 799位核苷酸T→A的转换，导致蛋白质产物中第600位的缬氨酸（V）被谷氨酸（E）替代^[8]^，常见于甲状腺瘤、直肠癌及黑色素瘤患者。

## 5 *BRAF*基因突变与NSCLC

### 5.1 *BRAF*突变在NSCLC中的流行病学特点

NSCLC *BRAF*突变率约为3%^[8]^，其中大部分是腺癌。一项肺癌小鼠模型^[3]^表明，NSCLC中存在BRAF的V600E突变表达上调。近来一项研究^[9]^对697例肺腺癌患者进行*EGFR*、*KRAS*与*BRAF*突变检测及相关分析，记录包括患者年龄、性别、种族、肿瘤分期、治疗史及吸烟史等临床特征。其中使用基于质谱分析法的MassARRAY系统（Sequenom, San Diego, CA）对*BRAF*突变进行分析，识别了3种*BRAF*突变：V600E（外显子15）、G469A（外显子11）、D594G（外显子15）。与黑色素瘤的*BRAF*突变80%以上为V600E突变不同，NSCLC的*BRAF*突变主要为激酶结构域中D594G突变（11%, *n*=2）及在活化域中甘氨酸环G469A突变（39%, *n*=7）和V600E突变（50%, *n*=9）^[8]^。近年来研究^[10]^表明，BRAF蛋白可以独立于RAS蛋白而发生作用。*BRAF*基因突变与*EGFR*、*KRAS*等突变相互独立、相互排斥且不同时出现^[9, 11]^，在这18例*BRAF*突变的患者中未检测到*EGFR*、*KRAS*或*ALK*突变^[9]^，也佐证了这个说法。*BRAF*与*KRAS*基因突变可能发生于肿瘤发生的相似阶段，即始动后恶性转变前，这两种突变可能在肿瘤的发生作用中是等同的。在生物学上，*BRAF*突变会使得激酶活性增加，且能使MAPK2、MAPK3结构性活化。近年一项研究^[9]^提示，与*EGFR*、*KRAS*突变多发生于非吸烟肺癌患者不同，*BRAF*突变多发生于吸烟肺癌患者。

### 5.2 BRAF抑制剂

目前已经研发出许多BRAF抑制剂。第一代BRAF抑制剂索拉菲尼是首个已有临床试验的RAF激酶抑制剂，目前被FDA批准在肾细胞癌和肝细胞癌中使用^[5]^。它起初被单一认为是BRAF抑制剂，后来发现是CRAF、BRAF、血管内皮生长因子受体、酪氨酸激酶受体等多个激酶的抑制剂，缺乏选择性。同时索拉菲尼在有着最高*BRAF*突变率的黑色素瘤的治疗中缺乏有意义的临床活性。在肾癌治疗中，积累的经验也提示索拉菲尼仅用作抗血管生成，而缺乏临床上BRAF抑制剂的功效^[12]^。在NSCLC治疗中，索拉菲尼作为抗血管形成因子联合化疗使用^[13]^，同样也缺乏临床功效。第二代是以PLX4032为代表的小分子高选择性BRAF抑制剂，尤其在*BRAF*基因V600E突变黑色素瘤的Ⅲ期临床试验^[14]^中取得明显疗效。PLX4032有着高度选择性，特异性地抑制*BRAF*（V600E）突变型细胞ERK磷酸化作用，而对BRAF野生型无抑制作用^[14]^。在BRAF（V600E）突变黑色素瘤模型中以及其异种移植临床随访研究^[15, 16]^中，PLX4032被证实能够强有力地抑制BRAF信号传导，包括诱导细胞周期停滞以及细胞凋亡。另外，原发的及继发的PLX4032耐药问题有待研究，信号通路的转换或许是导致耐药的最可能因素。所以同时抑制多种不同通路的联合治疗或许能保证更好的疗效。虽然PLX4032的毒性反应很低，但是在黑色素瘤的治疗中其诱发角化棘皮瘤及皮肤鳞状细胞癌等副作用值得关注^[17]^。

*BRAF*在NSCLC中的突变率约为3%，其中半数是非V600E突变，提示NSCLC的*BRAF*突变从本质上与黑色素瘤不同。然后针对靶向治疗药物治疗黑色素瘤*BRAF*（V600E）突变研究已经比较深入和成熟，而在NSCLC中的疗效尚待完善。对*BRAF*（V600E）突变型细胞高度选择性的靶向药物尤其是PLX4032在NSCLC中的作用有待进一步研究，同时也应该注意研发其它高选择性的靶向治疗药物。

### 5.3 *BRAF*基因在NSCLC表皮生长因子受体酪氨酸激酶抑制剂（epidermal growth factor receptor-tyrosine kinase inhibitor, EGFR-TKI）治疗中的预测作用

目前*EGFR*突变已经被确立为评估EGFR-TKI（吉非替尼和厄洛替尼）治疗敏感性的有效预测指标，有效率高达70%^[18]^。一项针对未经治疗的转移性NSCLC患者的前瞻性随机对照Ⅲ期研究（Iressa Pan-Asia Study, IPASS）^[18]^发现，吉非替尼一线治疗比顺铂辅助化疗对*EGFR*突变阳性的肺癌患者拥有更长的无进展生存期。与*EGFR*突变预测TKI疗效阳性价值相反，*BRAF*突变预示抗EGFR靶向治疗的耐药性。癌基因*BRAF*能使细胞对EGFR-TKI产生耐药。检测BRAF临床功效的回顾性分析^[19]^表明西妥昔单抗及帕尼单抗对*BRAF*突变的患者无效。

## 6 总结

在肿瘤治疗进入基于分子标志物的“个体化”医学时代，需要对肿瘤细胞携带的复杂基因突变进行系统的分子分型。近年来对NSCLC研究热点，更多地着重于对基于特异的基因突变的NSCLC临床相关分子事件的进一步细分。*BRAF*突变在NSCLC中的意义越来越受到广大学者的关注。

2002年英国肿瘤基因组计划的科学家Davies等^[8]^对来自于530个不同肿瘤细胞株的基因组DNA进行筛查，首次在人体内证实有43株细胞发生了*BRAF*基因突变，且在所有肿瘤中的发生率是8%，而在欧洲人群中肺癌中是3%。在一项针对非白种人的研究^[20]^中，日本肺腺癌患者*BRAF*突变仅有1%。近年来研究提示癌基因*BRAF*能使细胞对EGFR-TKI产生耐药。

继续对*BRAF*基因突变进行深入研究可望为阐明NSCLC致病的分子机制以及寻找新的治疗方法提供思路，为研究NSCLC的分子靶向治疗提供理论依据。其对于NSCLC的进展及预后的意义尚不明确，其能否作为NSCLC TKI治疗耐药判断的有效手段，尚待进一步研究证实。
